# Particle Size Distribution and Predicted Lipid Bioaccessibility of Almonds and the Effect of Almond Processing: A Randomised Mastication Study in Healthy Adults

**DOI:** 10.3390/nu15030489

**Published:** 2023-01-17

**Authors:** Alice C. Creedon, Estella S. Hung, Eirini Dimidi, Terri Grassby, Sarah E. Berry, Kevin Whelan

**Affiliations:** 1 Department of Nutritional Sciences, King’s College London, London SE1 9NH, UK; 2 School of Biosciences, University of Surrey, Guildford GU2 7XH, UK

**Keywords:** almond, mastication, particle size distribution, lipid bioaccessibility, processing, whole foods, plant foods, digestion

## Abstract

Almonds are rich in unsaturated lipids, which play a role in some of the reported benefits of almond consumption for human health. Almond lipids are poorly bioaccessible due to almonds’ unique physicochemical properties that influence particle size distribution (PSD) following mastication, allowing much intracellular lipid to escape digestion in the upper gastrointestinal tract. To investigate the impact of commercial processing (grinding almonds into flour), on PSD and predicted lipid bioaccessibility following mastication, a randomised cross-over design mastication study was conducted in healthy adults. The PSDs of masticated whole and ground almonds was assessed using two laboratory methods (mechanical sieving and laser diffraction). PSD from mechanical sieving was used to calculate lipid bioaccessibility using a theoretical mathematical model. Thirty-one healthy adults (18–45 years) completed both mastication sessions. Following mastication, ground almonds had a PSD with significantly fewer larger particles and more smaller particles, compared with whole almonds. Predicted lipid bioaccessibility of masticated ground almonds (10.4%, SD 1.8) was marginally but significantly greater than the predicted lipid bioaccessibility of masticated whole almonds (9.3%, SD 2.0; *p* = 0.017). Commercial grinding of almonds significantly influences the PSD of almonds following mastication, which results in a modest but significant increase in predicted lipid bioaccessibility.

## 1. Introduction

Nuts are rich in fibre, polyphenols and unsaturated lipids [1,2], and have been consistently shown to benefit health by improving markers of metabolic [3] and cardiovascular risk [4,5]. Almonds (*Prunus dulcis*) are the most produced tree nut worldwide [6] and are consumed by 7.6% of the UK population [7]. The beneficial effects of almonds on cardiovascular, metabolic [8,9] and gastrointestinal health [10] have been attributed to their nutritional content and their unique food matrix. Almonds possess unique physicochemical properties that limit release of certain nutrients during human digestion, in particular almond lipids. For example, factors such as small cell size, the presence of tough cell walls and unique fracture properties that limit cell rupture, as well as storage of lipids in intracellular droplets, limit the release of intracellular lipids for digestion in the upper gastrointestinal tract [11,12,13].

The term “bioaccessibility” refers to the proportion of a nutrient released from a complex food matrix that is therefore potentially available for absorption in the gastrointestinal tract [11,14]. The bioaccessibility of lipids from almonds is dependent on particle size, which determines the proportion of ruptured cells in the almond tissue following mechanical breakdown by processing prior to consumption (e.g., chopping, grinding, milling) or mastication during consumption [15,16].

The impact of mastication and processing on particle size distribution (PSD) and lipid bioaccessibility has been demonstrated in experiments in which almonds were subjected to simulated mastication whereby almonds were first minced, to simulate the mechanical breakdown of food, and then mixed with salivary enzymes [16]. The measured lipid bioaccessibility of whole natural almonds subjected to this method was reported as only 8.9% of total lipids, in comparison to 94% from almond ground into butter [16]. This phenomenon is likely responsible for the Atwater factors overestimating the metabolizable energy (ME) of whole natural almonds (25% greater than when empirically measured), in comparison to almond butter whose ME as predicted by Atwater factors is the same as when empirically measured [17]. The processing of almonds into butter involves grinding almonds until smooth, resulting in almost complete cellular disruption and total release of intracellular lipids. Almonds are also available in a commercially ground form that is widely used in baking. To our knowledge, the impact of commercial processing of almonds by grinding on post-mastication PSD and lipid bioaccessibility has not been investigated experimentally.

The impact of commercial processing on almond lipid bioaccessibility has important implications for human health. A systematic review and meta-analysis including trials investigating the impact of nuts on gut microbiota and gut health related outcomes revealed almond specific effects on gut microbiota composition at the genus level, and α-diversity [10]. It has been hypothesised that almond lipids might play a role in the observed prebiotic effect of almonds on the gut microbiota [18]. When consuming whole almonds, lipids, along with intracellular proteins, carbohydrates and non-nutritive bioactives, escape digestion in the upper gastrointestinal tract and reach the colon, where they are available for utilisation by the microbiota [11].

A recent randomised controlled trial (RCT) [19] was designed to test the hypothesis that consumption of almonds results in a beneficial effect on faecal bifidobacteria, due to their content of readily fermentable fibre and polyphenols [11,20,21,22], and potentially, intracellular almond lipids. Two almond arms were included (whole natural almonds, commercially ground almonds). As almonds are composed of 50% lipids [1], which are stored in intracellular lipid droplets, all of which are protected by indigestible plant cell walls, it was hypothesised that grinding of almonds prior to consumption would increase lipid bioaccessibility for digestion. Therefore, in participants consuming ground almonds, fewer lipids would reach the colon, and impact the gut microbiota, in comparison to those consuming whole almonds. However, the results revealed no differences in gut microbiota composition between participants consuming whole in comparison to ground almonds, calling into question whether almond processing impacts PSD and nutrient bioaccessibility [19].

The PSD and nutrient bioaccessibility of almonds have not been extensively explored and the impact of commercial grinding of almonds on PSD and lipid bioaccessibility is unknown. The majority of studies reporting health benefits of almonds have tested the effects of whole almonds, and attributed benefits to nutrient content of almonds. Thus, the investigation of nutrient bioaccessibility following processing may have important implications for human health. 

We therefore performed a randomised mastication study with the aim to: (a) measure and compare the PSDs of masticated whole and ground almonds, using two different techniques; and (b) compare the predicted lipid bioaccessibility of masticated whole and ground almonds.

## 2. Materials and Method

### 2.1. Study Design

This study was a randomised, crossover design mastication study in healthy volunteers consisting of a single study visit with two mastication sessions. A mastication study is required as mastication has a key role in reducing PSD and reflects the presentation of almonds as they appear in the gut in vivo. It has been demonstrated that there is large inter-individual variability in masticatory parameters such as the number and duration of mastication cycles, and that PSDs are dependent on food type [23]. 

Therefore, a within-subjects design was determined to be most appropriate to compare the PSDs and lipid bioaccessibility of whole and ground almonds. Randomisation was performed by an independent researcher, using the online randomisation website sealedenvelope.com (Sealed Envelope Ltd. 2020, London, UK). Participants were assigned to one of two sequences (whole almonds followed by ground almonds; ground almonds followed by whole almonds) in a 1:1 ratio. The sequence was concealed from researchers in sealed envelopes that were opened at the mastication study visit. Mastication sessions took place at the Metabolic Research Unit, King’s College London.

### 2.2. Participants

Participants were those who had previously completed an RCT investigating the impact of almond consumption on gut health [19]. Briefly, eligible participants were healthy men and women, aged 18–45 years with a BMI ≥18.5 kg/m^2^ and ≤29.9 kg/m^2^ and no allergy, intolerance or dislike of almonds or current infectious disease. Ethical approval was granted by the King’s College London Research Ethics Committee (HR-17/18-5341). The procedures of the trial were documented in a protocol published on clinicaltrials.gov (accessed on 8 January 2021) (NCT03581812) prior to commencement of recruitment.

### 2.3. Collection of Mastication Samples

Incomplete dentition, presence of dentures/false teeth and/or recent dental treatment were considered as confounding factors that may impact (1) normal masticatory behaviour due to pain or discomfort in part of the mouth or (2) particle recovery due to lodgement of particles in oral crevices. These data were collected from participants prior to study visit.

Whole and ground almonds were provided by the Almond Board of California. To facilitate consumption, ground almonds were mixed with water to form a paste prior to mastication (2.7 mL water was added to each 5 g aliquot of ground almond). In order to record the number of mastication cycles taken by each participant, mastication and swallowing were observed separately for each almond form prior to sample collection. Briefly, participants brushed their teeth before chewing two aliquots of each test food (either whole or ground almonds; random order; 5 g per aliquot) under the observation of a study researcher who counted the number of mastication cycles (chews) taken prior to swallowing. Mean number of mastication cycles for each almond form was calculated.

During the mastication experiment, participants were asked to chew (for the mean number of cycles calculated previously) three pre-weighed aliquots of either whole or ground almonds (approx. 5 g each) and without swallowing any almond were asked to expectorate all aliquots into the same pre-weighed beaker fitted with a 20 μm nylon mesh. Participants rinsed their mouth with 25 mL water after each aliquot and expectorated into the same beaker to maximise recovery of almond particles from the mouth. Participants brushed their teeth once again and the session was repeated for the second test food.

Beakers were allowed to stand for 20 min to allow saliva and rinse water to pass through the 20 μm nylon mesh. The nylon mesh was inserted into a falcon tube, and centrifuged at 400× *g* for 2 min to maximise removal of liquids from the sample. Masticated almonds were then transferred from the mesh into falcon tubes for later analysis by mechanical sieving (approx. 9 g), and laser diffraction (approx. 2 g). Samples were snap frozen on dry ice and stored at −80 °C.

### 2.4. Mechanical Sieving

Samples were thawed at 4 °C and loaded onto a stack of pre-weighed ultra-sonically cleaned sieves (Endecotts Ltd., London, UK) with the following aperture sizes from top to bottom: 3350, 2000, 1000, 500, 250, 125, 63 and 45 µm, a 20 µm nylon mesh and a sieve base. The sample was washed with deionised water before being placed on a mechanical sieve shaker (Endecotts Ltd., UK) for 15 min to allow the masticated almonds particles to pass through the sieves until they reached the sieve with an aperture size smaller than the size of that particle. Sieves were washed with deionised water to facilitate movement of lodged particles down through the sieve tower. To remove all water, leaving only the masticated almond samples, all sieves were then placed in a forced air oven at 56 °C for 24 h after which each sieve was weighed at 15 min intervals until a constant weight was reached (within 0.1 g). Oven temperature was increased to 80 °C and sieve bases were dried for a further 12 h, after which each sieve was weighed at 15 min intervals until weight was constant.

For PSD analysed by mechanical sieving, the proportion of masticated almonds retained on each sieve was expressed as a percentage of the total weight of sample recovered from the sieves (% weight; not including the contents of the sieve base).

### 2.5. Laser Diffraction

Samples were thawed at 4 °C. Particles >2000 µm and <20 µm were removed by mechanical sieving prior to analysis to avoid interference with the laser diffraction instrument as described previously [15]. The sample was mixed with a small amount of deionised water to facilitate separation of the particles and divided into approximately equal aliquots. 

Laser diffraction was performed on a Mastersizer 2000 instrument fitted with a Hydro 2000 G dispersant unit (Malvern Panalytical Ltd., Malvern, UK). The settings on the MasterSizer were as follows: pump speed, 700; stirrer speed, 1175; ultrasound, 70 [15]. Individual aliquots were poured into the bath until the laser obscuration was between 10 and 15%. The software recorded three consecutive 10-s measurements for each aliquot and generated an average of the three measurements. All readings were stored in the MasterSizer online software (Version 6.1, Malvern Panalytical Ltd., Malvern, UK).

For PSD analysed by laser diffraction, mean PSD at each particle size interval was calculated from individual aliquots. The proportion of the sample in each particle size interval was expressed as a percentage of the total sample volume (% volume).

### 2.6. Predicted Lipid Bioaccessibility

A theoretical model for predicting lipid bioaccessibility in almonds has been developed previously [12], and validated for use against empirically measured lipid bioaccessibility by the Soxhlet procedure [15,16]. PSDs of whole and ground almonds were analysed by mechanical sieving as described above and these data were used in the equation:

Lipids released = ½ [(64/π^2^) (d/p) − 8 (d/p)^2^ + 4/3π (d/p)^3^] (1)

where d is the mean diameter of almond cells as measured previously (36 µm) [12], and p is the equivalent cubic particle size at that size fraction [12]. The model calculated predicted lipid bioaccessibility for each particle size fraction assessed (i.e., each sieve aperture size). Total lipid bioaccessibility for a sample was predicted by multiplication of these values by the % weight of almond recovered at each fraction for that sample. The model can also be used to calculate the threshold particle size value (P), which is the value for particle size at which no more intact cells are present, indicating complete intracellular lipid release.

### 2.7. Statistical Analysis

Data were analysed on IBM SPSS Statistics (version 26; IBM, Portsmouth, UK). Descriptive statistics were used to summarise demographic characteristics and outcome data. For continuous outcomes, mean and standard deviation were calculated.

Summary data for PSD were presented as mean % weight (mechanical sieving) or mean % volume (laser diffraction) and standard deviation (SD). To assess inter-individual variation at each level of particle size, a coefficient of variation (CV) was calculated for each sieve aperture size within each almond type using the formula: CV (%) = (SD/mean) × 100.

Before analysis, all continuous raw data were checked for normality and outliers using Q-Q plots and the Shapiro–Wilk test. Student’s paired *t*-test was used to assess differences in masticatory parameters and predicted lipid bioaccessibility between whole almonds and ground almonds. Differences in PSDs were assessed by two-way repeated measures ANOVA with sieve aperture size and almond form as factors. Where there was a significant interaction, simple main effects were analysed at each particle size fraction (Student’s paired *t*-test) and *p*-values were corrected for multiple comparisons (Bonferroni). *p*-values of <0.05 were considered statistically significant for all tests.

## 3. Results

### 3.1. Particpant Characteristics

**Figure 1:**
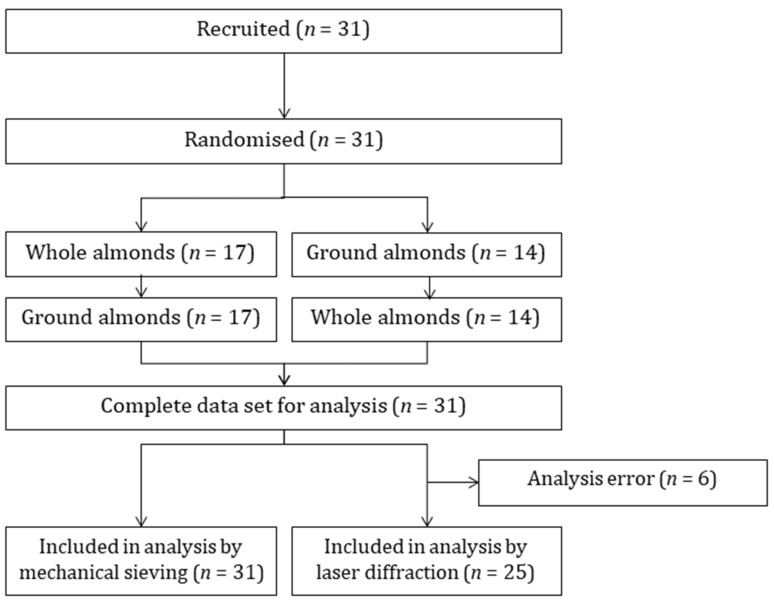
Consort diagram.

The consort diagram is presented in Figure 1. A total of 31 participants were recruited to the mastication trial and all participants completed both mastication sessions. A technical error during analysis of 6 samples meant that data for laser diffraction were only available for 25 participants. Demographic characteristics are presented in Table 1.

### 3.2. Masticatory Parameters

Data were gathered on each participant’s dentition, the results of which are presented in Table 2. The numbers of mastication cycles required for complete mastication of whole almonds (53, SD 22.5, range 26–125) were significantly greater than that for ground almonds (21, SD 11.7, range 5–52; *p* < 0.001; paired *t*-test).

### 3.3. Particle Size Distribution Assessed by Mechanical Sieveing

**Figure 2:**
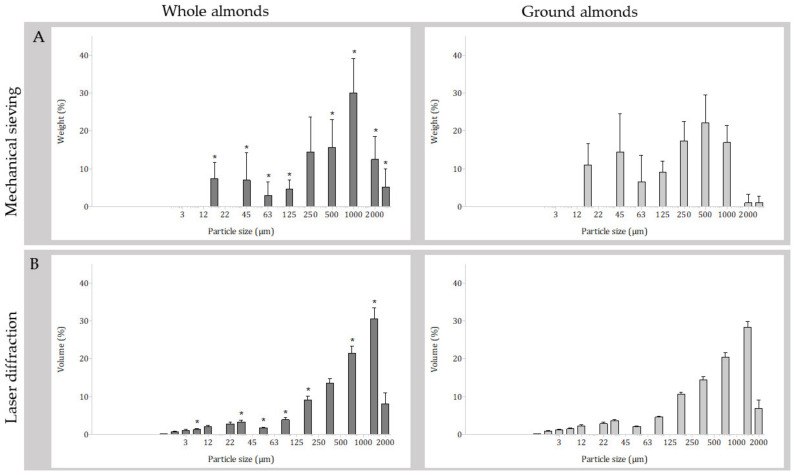
Particle size distributions of masticated whole and ground almonds (**A**) measured by mechanical sieving; bars are mean weight recovered; *n* = 31 participants provided paired data; and (**B**) measured by laser diffraction; bars are mean volume; *n* = 25 participants provided paired data. Error bars are standard deviation; (*) indicates significant difference between almond forms for that method (*p* < 0.05).

The mean total weight of particles recovered was significantly different (*p* = 0.038) between masticated whole almonds (12.4 g, SD 1.7) and ground almonds (11.6 g, SD 1.8). The PSD of masticated whole and ground almonds as assessed by mechanical sieving is illustrated in Figure 2a and Table 3. Following repeated measures ANOVA, the PSDs (% weight) of masticated whole and ground almonds assessed by mechanical sieving revealed a statistically significant interaction between almond form (whole or ground) and particle size (sieve aperture size) on PSD (*p* < 0.001). Simple main effects for nut type revealed significantly more particles retained for masticated ground almonds in comparison to masticated whole almonds in the smaller aperture sieves at 20 µm (*p* = 0.009), 45 µm (*p* = 0.018), 63 µm (*p* < 0.001), 125 µm (*p* < 0.001) and 500 µm (*p* < 0.001) and significantly more particles retained for masticated whole almonds in comparison to masticated ground almonds in the larger aperture sieves at 1000 µm (*p* < 0.001), 2000 µm (*p* < 0.001) and 3350 µm (*p* < 0.001).

### 3.4. Particle Size Distribution Assessed by Laser Diffraction

The PSD of masticated whole and ground almonds as assessed by laser diffraction is illustrated in Figure 2b and Table 4. Following repeated measures ANOVA, the PSDs (% volume) of masticated whole and ground almonds assessed by laser diffraction revealed a statistically significant interaction between almond form (whole or ground) and particle size fraction on PSD (*p* < 0.001). Simple main effects for nut type revealed significantly more masticated ground almonds particles were measured at the smaller range of <6.2 µm, <42.4 µm, <54.0 µm, <80.5 µm, and <153.1 µm (all *p* < 0.001) in comparison to masticated whole almonds. Meanwhile, there were more masticated whole almond particles measured at the larger range of <553.4 µm and <1052.0 µm (both *p* = 0.028) in comparison to masticated ground almonds.

### 3.5. Predicted Lipid Bioaccessibility

Lipid bioaccessibility of masticated whole and ground almonds was predicted using the theoretical model with data obtained from particle size analysis by mechanical sieving (*n* = 31). Predicted lipid bioaccessibility from masticated ground almonds (10.4%, SD 1.8) was slightly but significantly greater than that of masticated whole almonds (9.3%, SD 2.0; *p* = 0.017).

The model indicated a threshold particle size value (P) of approximately 54 µm for almonds. Thus, to obtain complete lipid release, all particles would need to be smaller than 54 µm. Masticated whole almond samples contained significantly fewer particles <54 µm (11.5%, SD 1.7) in comparison to masticated ground almonds (12.6%, SD 1.5; *p* = 0.003; paired samples *t*-test using data from laser diffraction).

## 4. Discussion

This mastication study was conducted to test the hypotheses that mastication of commercially ground almonds would result in a PSD with smaller particles in comparison to whole almonds, and that this would influence subsequent predicted lipid bioaccessibility. Our results support the above hypotheses; there were differences in PSDs in which masticated ground almonds had significantly more particles <150 µm, and masticated whole almonds had significantly more particles >1000 µm. This resulted in a modest, but significantly greater predicted lipid release from masticated ground almonds, in comparison to masticated whole almonds.

We confirm the results of previous studies demonstrating that following mastication whole almonds have a wide PSD, including many larger particles that prevent complete lipid release during digestion, with one study reporting masticated whole almonds containing 35–40% of particles >500 µm [15].

There were significant differences in the number of mastication cycles required for whole and ground almonds. This variability is supported by previous suggestions that masticatory performance is determined by the requirement that a food bolus reaches a precisely determined texture before it can be swallowed [24]. In the current study whole almonds underwent 2.5× as many chews as ground almonds, to achieve the optimal food bolus texture. Palatability is also known to impact chewing behaviour, whereby increased palatability is associated with decreased mastication cycles per unit of food [25]. Despite the considerably increased mastication of whole almonds, masticated ground almonds still have smaller particle sizes, indicating that grinding of almonds results in greater mechanical disruption than can be achieved through mastication.

It is important to note the significant inter-individual variability in PSDs following mastication as indicated by large CVs (ranging from 0–100% for laser diffraction and 27–154% for mechanical sieving; Table 3 and Table 4). This contrasts with a previous study which investigated mastication of various plant foods and reported low inter-individual variation in PSDs of masticated nuts (pistachios, almonds, peanuts), but did not report CVs [24]. It is widely accepted that mastication and chewing behaviour varies widely among healthy humans, and therefore this variation is unsurprising [26,27].

Interestingly, the PSD profile of masticated ground almonds appeared to be bimodal when assessed using both measurement methods, with a peak in the proportion of particles recovered on the 45 µm sieve and the corresponding level of particle size for laser diffraction, and another larger peak in proportion of particles recovered on the 500 µm sieve or around the 1000 µm level for laser diffraction. This was not the case for whole almonds, which had a peak in proportion of particles recovered on the 1000 µm sieve only. This potentially indicates an uneven distribution of particle sizes following the grinding process, whereby certain particle sizes are over-represented following grinding and mastication has no further effect at these sizes. This theory is supported by research reporting that the granularity of a food during mastication must reach a predetermined state before swallowing is initiated, and that this is achieved by a highly individualised chewing strategy [23].

This was the first study to analyse the lipid bioaccessibility from masticated commercially ground almonds. Predicted lipid bioaccessibility from masticated whole almonds was 9.3%, which agreed with previous analyses of lipid release from masticated whole almonds measured by Soxhlet extraction (7.8–11.1%) and predicted using the theoretical model (9.6%) [16,28]. Despite a significantly greater proportion of small particles in the PSD of ground almonds in comparison to whole almonds, the difference in lipid bioaccessibility between these almond forms was modest (mean difference 1.1%, SD 2.3). A previous RCT was conducted to investigate the effect of whole and ground almonds on gut microbiota and gut metabolism, based on the assumption that differences in nutrient bioaccessibility would drive differences in action in the gut [19]. Although almonds impacted the abundance of several bacteria at the genus level, as well as butyrate concentrations, there were no differences in microbiota composition between whole almond and ground almond groups [19]. Findings from the current study suggest that, despite differences in PSD profiles, commercial grinding does not result in appreciable differences in nutrient bioaccessibility capable of altering gut microbiota composition.

Bioaccessibility is only one factor that has an impact on the almond lipids available for absorption. Factors such as total fatty acid content of different almond harvests and method of storage will also influence the almond lipids available for absorption and their subsequent impact on human health [29]. Our study measures the theoretical lipid release from masticated whole and processed nuts. However, we did not measure the impact of total fatty acid content and storage method on this, which is likely affected by lipid oxidation over time [30]. The impact of pre-ingestive factors (e.g., agricultural conditions, harvest, storage) on subsequent health effects of nuts should be investigated, together with the effect of almond processing, as has been investigated here.

The results of the current study are not representative of the impact of later stages of digestion on lipid release from almond cells. The impact of enzymatic digestive processes in the stomach and duodenum on lipid release from almond cells would be technically difficult to measure in humans, and would require consumption of almonds in isolation over a prolonged period, a method that would have considerable practical and ethical implications [31]. One study investigating lipid release during upper gastrointestinal digestion reported that masticated whole almonds released significant amounts of lipids during both simulated gastric (16.4%) and simulated duodenal (32.2%) digestion [28]. Additionally, significant increases in faecal lipid excretion have been reported in participants who were asked to masticate almonds minimally (10 mastication cycles; 43.7% ingested lipid excreted) in comparison to those asked to masticate almonds extensively (40 mastication cycles; 30.8% ingested lipid excreted) [32]. Taken together, these findings indicate that despite continued breakdown of almond cells, and release of intracellular lipid during upper GI digestion, significant amounts of cells, and their intracellular lipid droplets, remain intact and are lost to digestion.

In the current study, PSD data from analysis of masticated almonds by mechanical sieving was used to predict lipid bioaccessibility using a theoretical model [12]. While the model was validated using PSD data from analysis of masticated whole almonds by mechanical sieving in combination with laser diffraction, the use of mechanical sieving alone was considered appropriate due to its ability to measure particle sizes over a wide range (20–3350 µm). In comparison, laser diffraction has a maximum measurement limit of 2000 µm, although it does have greater granularity at very small particles sizes <20 µm (at which point the physiological consequences are likely minimal). Previous studies have attributed low lipid bioaccessibility of masticated whole almonds to the high proportion of larger particles [15]. Large particles (>500 µm) have a crucial effect on reducing bioaccessibility due to their low surface area/volume ratio, resulting in the majority of cells in these particles remaining intact. While combining the data from mechanical sieving and laser diffraction would give more detailed information on particle size fractions at lower size ranges, this information might be considered less physiologically relevant when considering the impact of larger particles on lipid release for digestion. In addition, this combination might give rise to error due to the measurement of particle size using different metrics in the two methods (i.e., % weight; % volume).

We have demonstrated that both whole and ground almonds have PSDs that prevent complete lipid release during the first stage of digestion (mastication), and that this would likely result in lower metabolizable energy than predicted by Atwater factors when consuming these forms of almond. This has important implications for human health. In groups that could benefit from increased energy consumption, for example for recovery following injury, or in the elderly, it might be recommended that consuming almonds in a processed form (ground almonds, almond butter) is beneficial. Conversely, our findings support the evidence that despite their high lipid content and energy density, whole almonds can be added to the diets of those attempting weight loss without impacting this outcome [33].

To our knowledge this is the first trial to investigate the impact of commercial grinding on the PSD and predicted lipid bioaccessibility of almonds. Limitations of the trial include storage of almonds prior to particle size analysis, which may result in further breakdown of almond particles due to the action of salivary enzymes, together with the mechanical forces of freezing. To limit these effects, masticated almond samples were snap frozen on dry ice prior to storage at −80 °C. At such temperatures, salivary enzymes are no longer active.

It was not possible to calculate the total % recovery of the original weight of almonds that were masticated due to (1) separation of the masticated almond samples into aliquots for analysis; and (2) the contrasting outcome data of the methods (sieving—% weight; laser diffraction—% volume). Mastication sessions and sample collection were conducted based on previously published research, that reported 85.4% (SD 1.5%) recovery following mastication of whole natural almonds [15]. As in previous studies, recovery was maximised by oral gavage with water following mastication. Due to the comparability of methods between previous research and the findings reported here, we expected similar recovery for whole almonds in this study. In our study, recovery of particles from sieves was significantly higher for whole almonds in comparison to ground almonds (mean difference 1.2 g, 95% CI 0.3, 2.0). It can be hypothesised that due to reduced particle size of ground almonds, significantly more ground almond particles were not recovered following mastication due to lodgement in oral crevices. However, it is not possible to identify whether the particle sizes of recovered almond particles (as reported here) differed from those not recovered from the mouth.

## 5. Conclusions

In conclusion, we confirm that mastication does not provide sufficient mechanical disruption to result in a PSD that facilitates complete lipid release from whole almonds. In addition, commercial processing of almonds by grinding results in a post-mastication PSD with a larger proportion of smaller particles, and a subsequent modest increase in predicted lipid bioaccessibility compared to the results following mastication of whole almonds. However, it is unlikely that the difference in lipid bioaccessibility is sufficient to result in clinically meaningful differences to human health outcomes, for example gut microbiota composition. Future studies are needed to investigate the impact of alternative almond processing methods, for example grinding of almonds into butter (almost complete lipid release, but also change in cell matrix [34]) on PSD and predicted lipid bioaccessibility, and the consequences for human health.

## Figures and Tables

**Table 1:** Demographic characteristics of participants.

	Total Sample (*n* = 31)
Age (years), mean (SD)	27.2 (6.2)
Female, *n* (%)	28 (90.3)
Body mass index (kg/m^2^), mean (SD)	22.7 (2.9)

**Table 2:** Participant dentition.

	Yes	No
Do you have all your teeth? ^a^	21/25 (84.0)	4/25 (16.0)
Do you have any false teeth/dentures?	0 (0.0)	27 (100.0)
Have you had any dental treatment in the past month?	2 (7.4)	25 (92.6)
Do you have any mouth/chewing problems?	0 (0.0)	27 (100.0)
Do you experience considerable bleeding when brushing your teeth?	0 (0.0)	27 (100.0)

Data are number, *n* (%); Total *n* = 27, except where indicated; ^a^ *n* = 25.

**Table 3:** Particle size distributions of masticated whole and ground almonds assessed by the mechanical sieving technique.

	Amount at Each Particle Size (% Weight), Mean (SD)				Coefficient of Variation between Participants at Each Particle Size, CV %	
Sieve Aperture Size (µm)	Whole Almonds	Ground Almonds	Mean Difference	*p*-Value ^a^	Whole Almonds	Ground Almonds
20	7.4 (4.2)	11.0 (5.6)	−3.7 (−5.6 to −1.7)	0.009	56.7	50.9
45	7.0 (7.3)	14.5 (10.0)	−7.5 (−12.0 to −3.1)	0.018	104.3	69.0
63	3.0 (3.6)	6.6 (7.0)	−3.6 (−5.0 to −2.2)	<0.001	120	106.1
125	4.7 (2.3)	9.1 (2.9)	−4.4 (−5.7 to −3.1)	<0.001	48.9	31.9
250	14.5 (9.2)	17.4 (5.1)	−3.0 (−6.3 to 0.4)	1	63.4	29.3
500	15.7 (7.3)	22.2 (7.3)	−6.6 (−9.8 to −3.3)	<0.001	46.5	32.9
1000	30.1 (9.1)	16.9 (4.6)	13.1 (9.6 to 16.6)	<0.001	30.2	27.2
2000	12.5 (6.1)	1.0 (2.2)	11.5 (9.3 to 13.7)	<0.001	48.8	220
>3350	5.2 (4.8)	1.1 (1.7)	4.1 (2.1 to 6.1)	<0.001	92.3	154.5

Values are mean (SD) or mean difference (95% confidence interval); *n* = 31 participants provided paired data; CV. Coefficient of variation; Significant interaction between almond form (whole, ground) and sieve aperture size from two-way repeated measures ANOVA (*p* < 0.001); ^a^ *p*-values are the result of simple main effects for nut type with Bonferroni correction for multiple comparisons.

**Table 4:** Particle size distributions of masticated whole and ground almonds assessed by laser diffraction.

	Amount at Each Particle Size (% Volume), Mean (SD)				Coefficient of Variation between Participants at Each Particle Size, CV %	
Particle Size (µm)	Whole Almonds	Ground Almonds	Mean Difference	*p*-Value ^a^	Whole Almonds	Ground Almonds
<0.5	0.0 (0.0)	0.0 (0.0)	0	1	0.0	0.0
<0.9	0.1 (0.1)	0.2 (0.0)	−0.0 (−0.0 to 0.0)	0.784	100.0	0.0
<1.7	0.7 (0.2)	0.8 (0.2)	−0.1 (−0.1 to 0.0)	1	28.6	25.0
<3.2	1.1 (0.2)	1.2 (0.2)	−0.1 (−0.2 to 0.0)	0.126	55	16.7
<6.2	1.3 (0.2)	1.5 (0.2)	−0.1 (−0.3 to −0.1)	<0.001	15.4	13.3
<11.7	2.1 (0.3)	2.3 (0.3)	−0.1 (−0.3 to −0.0)	0.602	14.3	13.0
<22.3	2.8 (0.4)	2.9 (0.4)	−0.1 (−0.2 to 0.1)	1	14.3	13.8
<42.4	3.3 (0.5)	3.7 (0.3)	−0.4 (−0.6 to −0.2)	<0.001	15.2	8.1
<54.0	1.8 (0.2)	2.1 (0.1)	−0.3 (−0.3 to −0.1)	<0.001	11.11	4.8
<80.5	3.9 (0.5)	4.6 (0.3)	−0.7 (−0.9 to −0.4)	<0.001	12.8	6.5
<153.1	9.2 (1.0)	10.6 (0.6)	−1.4 (−1.9 to −0.9)	<0.001	10.9	5.7
<291.1	13.6 (1.2)	14.4 (0.8)	−0.8 (−1.4 to −0.2)	0.056	8.8	5.6
<553.4	21.5 (1.8)	20.4 (1.2)	1.0 (0.4 to 1.7)	0.028	8.4	5.9
<1052.0	30.5 (2.9)	28.3 (1.5)	2.2 (0.9 to 3.4)	0.028	9.5	5.3
≤2000.0	8.0 (3.0)	6.9 (2.3)	1.1 (−0.6 to 2.7)	1	47.5	33.3

Values are mean (SD) or mean difference (95% confidence interval); *n* = 25 participants provided paired data; CV. Coefficient of variation; significant interaction between almond form (whole, ground) and sieve aperture size from two-way repeated measures ANOVA (*p* < 0.001); ^a^ *p*-values are the result of simple main effects for nut type with Bonferroni correction for multiple comparisons.

## Data Availability

Data available on request. The data presented in this study are available on request from the corresponding author. The data are not publicly available due to nature of consent obtained from participants.

## References

[1] Public Health England McCanse and Widdowson’s Composition of Foods Integrated Dataset (CoFID). https://www.gov.uk/government/publications/composition-of-foods-integrated-dataset-cofid.

[2] Rothwell J.A., Perez-Jimenez J., Neveu V., Medina-Remón A., M’hiri N., García-Lobato P., Manach C., Knox C., Eisner R., Wishart D.S. (2013). Phenol-Explorer 3.0: A Major Update of the Phenol-Explorer Database to Incorporate Data on the Effects of Food Processing on Polyphenol Content. Database.

[3] Tindall A.M., Johnston E.A., Kris-Etherton P.M., Petersen K.S. (2019). The Effect of Nuts on Markers of Glycemic Control: A Systematic Review and Meta-Analysis of Randomized Controlled Trials. Am. J. Clin. Nutr..

[4] Sabaté J., Oda K., Ros E. (2010). Nut Consumption and Blood Lipid Levels: A Pooled Analysis of 25 Intervention Trials. Arch. Intern. Med..

[5] del Gobbo L.C., Falk M.C., Feldman R., Lewis K., Mozaffarian D. (2015). Effects of Tree Nuts on Blood Lipids, Apolipoproteins, and Blood Pressure: Systematic Review, Meta-Analysis, and Dose-Response of 61 Controlled Intervention Trials. Am. J. Clin. Nutr..

[6] International Nut and Dried Fruit Council (2019). Nuts & Dried Fruits: Statistical Yearbook 2018/2019.

[7] Dikariyanto V., Berry S.E., Francis L., Smith L., Hall W.L. (2020). Whole Almond Consumption Is Associated with Better Diet Quality and Cardiovascular Disease Risk Factors in the UK Adult Population: National Diet and Nutrition Survey (NDNS) 2008–2017. Eur. J. Nutr..

[8] Kalita S., Khandelwal S., Madan J., Pandya H., Sesikeran B., Krishnaswamy K. (2018). Almonds and Cardiovascular Health: A Review. Nutrients.

[9] Berryman C.E., West S.G., Fleming J.A., Bordi P.L., Kris-Etherton P.M. (2015). Effects of Daily Almond Consumption on Cardiometabolic Risk and Abdominal Adiposity in Healthy Adults with Elevated LDL-Cholesterol: A Randomized Controlled Trial. J. Am. Heart Assoc..

[10] Creedon A.C., Hung E.S., Berry S.E., Whelan K. (2020). Nuts and Their Effect on Gut Microbiota, Gut Function and Symptoms in Adults: A Systematic Review and Meta-Analysis of Randomised Controlled Trials. Nutrients.

[11] Ellis P.R., Kendall C.W.C., Ren Y., Parker C., Pacy J.F., Waldron K.W., Jenkins D.J.A. (2004). Role of Cell Walls in the Bioaccessibility of Lipids in Almond Seeds. Am. J. Clin. Nutr..

[12] Grassby T., Picout D.R., Mandalari G., Faulks R.M., Kendall C.W.C., Rich G.T., Wickham M.S.J., Lapsley K., Ellis P.R. (2014). Modelling of Nutrient Bioaccessibility in Almond Seeds Based on the Fracture Properties of Their Cell Walls. Food Funct..

[13] Trombetta D., Smeriglio A., Denaro M., Zagami R., Tomassetti M., Pilolli R., de Angelis E., Monaci L., Mandalari G. (2020). Understanding the Fate of Almond (Prunus Dulcis (Mill.) D.A. Webb) Oleosomes during Simulated Digestion. Nutrients.

[14] Stahl W., van den Berg H., Arthur J., Bast A., Dainty J., Faulks R.M., Gärtner C., Haenen G., Hollman P., Holst B. (2002). Bioavailability and Metabolism. Mol. Aspects Med..

[15] Grundy M.M.L., Grassby T., Mandalari G., Waldron, Butterworth P., Berry S.E.E., Ellis P.R. (2015). Effect of Mastication on Lipid Bioaccessibility of Almonds in a Randomized Human Study and Its Implications for Digestion Kinetics, Metabolizable Energy, and Postprandial Lipemia. Am. J. Clin. Nutr..

[16] Mandalari G., Parker M., Grundy M., Grassby T., Smeriglio A., Bisignano C., Raciti R., Trombetta D., Baer D., Wilde P. (2018). Understanding the Effect of Particle Size and Processing on Almond Lipid Bioaccessibility through Microstructural Analysis: From Mastication to Faecal Collection. Nutrients.

[17] Gebauer S.K., Novotny J.A., Bornhorst G.M., Baer D.J. (2016). Food Processing and Structure Impact the Metabolizable Energy of Almonds. Food Funct..

[18] Liu Z., Lin X., Huang G., Zhang W., Rao P., Ni L. (2014). Prebiotic Effects of Almonds and Almond Skins on Intestinal Microbiota in Healthy Adult Humans. Anaerobe.

[19] Creedon A.C., Dimidi E., Hung E.S., Rossi M., Probert C., Grassby T., Miguens-Blanco J., Marchesi J.R., Scott S.M., Berry S.E. (2022). The Impact of Almonds and Almond Processing On Gastrointestinal Physiology, Luminal Microbiology and Gastrointestinal Symptoms: A Randomized Controlled Trial and Mastication Study. Am. J. Clin. Nutr..

[20] Mandalari G., Tomaino A., Arcoraci T., Martorana M., Lo Turco V., Cacciola F., Rich G., Bisignano A., Saija A., Dugo P. (2010). Characterization of Polyphenols, Lipids and Dietary Fibre from Almond Skins (*Amygdalus communis* L.). J. Food Compos. Anal..

[21] Bolling B.W. (2017). Almond Polyphenols: Methods of Analysis, Contribution to Food Quality, and Health Promotion. Compr. Rev. Food Sci. Food Saf..

[22] Dourado F., Barros A., Mota M., Coimbra M.A., Gama F.M. (2004). Anatomy and Cell Wall Polysaccharides of Almond (*Prunus dulcis* D. A. Webb) Seeds. J. Agric. Food Chem..

[23] Mishellany A., Woda A., Labas R., Peyron M.-A. (2006). The Challenge of Mastication: Preparing a Bolus Suitable for Deglutition. Dysphagia.

[24] Peyron M.-A., Mishellany A., Woda A. (2004). Particle Size Distribution of Food Boluses after Mastication of Six Natural Foods. J. Dent. Res..

[25] Frecka J.M., Hollis J.H., Mattes R.D. (2008). Effects of Appetite, BMI, Food Form and Flavor on Mastication: Almonds as a Test Food. Eur. J. Clin. Nutr..

[26] Pereira L.J., Duarte Gaviao M.B., van der Bilt A. (2006). Influence of Oral Characteristics and Food Products on Masticatory Function. Acta Odontol. Scand..

[27] Maeda R., Takei E., Ito K., Magara J., Tsujimura T., Inoue M. (2020). Inter-Individual Variation of Bolus Properties in Triggering Swallowing during Chewing in Healthy Humans. J. Oral. Rehabil..

[28] Mandalari G., Grundy M.M.-L., Grassby T., Parker M.L., Cross K.L., Chessa S., Bisignano C., Barreca D., Bellocco E., Laganà G. (2014). The Effects of Processing and Mastication on Almond Lipid Bioaccessibility Using Novel Methods of in Vitro Digestion Modelling and Micro-Structural Analysis. Br. J. Nutr..

[29] Yada S., Huang G., Lapsley K. (2013). Natural Variability in the Nutrient Composition of California-Grown Almonds. J. Food Compos. Anal..

[30] Luo K.K., Huang G., Mitchell A.E. (2022). Acceleration of Lipid Oxidation in Raw Stored Almond Kernels in Response to Postharvest Moisture Exposure. J. Sci. Food Agric..

[31] Grassby T., Mandalari G., Grundy M.M.-L., Edwards C.H., Bisignano C., Trombetta D., Smeriglio A., Chessa S., Ray S., Sanderson J. (2017). In Vitro and in Vivo Modeling of Lipid Bioaccessibility and Digestion from Almond Muffins: The Importance of the Cell-Wall Barrier Mechanism. J. Funct. Foods.

[32] Cassady B.A., Hollis J.H., Fulford A.D., Considine R.V., Mattes R.D. (2009). Mastication of Almonds: Effects of Lipid Bioaccessibility, Appetite, and Hormone Response. Am. J. Clin. Nutr..

[33] Mattes R.D., Kris-Etherton P.M., Foster G.D. (2008). Impact of Peanuts and Tree Nuts on Body Weight and Healthy Weight Loss in Adults. J. Nutr..

[34] Karabagias I.K., Karabagias V.K., Gatzias I., Riganakos K.A. (2018). Bio-Functional Properties of Bee Pollen: The Case of “Bee Pollen Yoghurt. Coatings.

